# An Improved DeepLabV3+-Based Method for Crop Row Segmentation and Navigation Line Extraction in Agricultural Fields

**DOI:** 10.3390/s26103142

**Published:** 2026-05-15

**Authors:** Letian Wu, Yongzhi Cui, Huifeng Shi, Xiaoli Sun, Jiayan Yang, Xinwei Cao, Ping Zou, Ya Liu

**Affiliations:** 1 Institute of Agricultural Equipment, Xinjiang Uygur Autonomous Region Academy of Agricultural Sciences, Urumqi 830091, China; 2 Xinjiang Key Laboratory of Intelligent Control Technology for Facility Agriculture, Urumqi 830091, China; 3 Chinese Academy of Agricultural Mechanization Sciences Group Co., Ltd., Beijing 100083, China; 4 State Key Laboratory of Agricultural Equipment Technology, Chinese Academy of Agricultural Mechanization Sciences, Beijing 100083, China

**Keywords:** DeeplabV3+, visual navigation, navigation line extraction, semantic segmentation

## Abstract

Accurate crop row detection is identified as a critical prerequisite for autonomous agricultural navigation, yet it remains challenging in complex field environments. To achieve a balance between segmentation accuracy, robustness, and real-time performance, an improved crop row segmentation and navigation method based on the DeepLabV3+ framework was developed. MobileNetV2 was adopted as the backbone to minimize computational costs, while feature representation was enhanced through integrated attention mechanisms and multi-scale fusion. Specifically, split-attention convolution was integrated into the backbone, a DenseASPP + SP module was employed for multi-scale contextual capture, and a Convolutional Block Attention Module (CBAM) was added to refine feature responses. Experimental results demonstrated that the proposed method outperformed mainstream models, achieving a mean Intersection over Union (mIoU) of 93.42% and an *f*1-score of 96.8%. The model maintained a lightweight architecture with 8.35 M parameters and a real-time speed of 32 FPS. Furthermore, crop row anchor points were extracted and processed via DBSCAN clustering and RANSAC fitting to generate high-precision navigation lines. Validation showed that the middle crop row yielded the highest fitting accuracy with minimal angular and lateral errors. This study provides an efficient visual perception solution for intelligent field operations.

## 1. Introduction

Precision agriculture is an advanced concept in the field of modern agriculture. It leverages scientific and technological advancements to transform traditional farming methods, thereby revolutionizing contemporary agriculture. Its aim is to enhance crop productivity while reducing costs and minimizing environmental impact. In this process, agricultural machinery is equipped with various sensors and advanced algorithms to achieve intelligence, enabling it to autonomously and efficiently perform a range of farm tasks such as planting, weeding, spraying, fertilizing, and harvesting [1]. However, intelligent agricultural machinery requires precise guidance during field operations, and automatic navigation and autonomous driving technologies have emerged as key solutions within agricultural machinery. Compared to navigation technologies such as the Global Navigation Satellite System (GNSS), Inertial Measurement Unit (IMU), and LiDAR, machine vision offers advantages such as its low cost, high resolution, and excellent perception of environmental structures [2]. Therefore, researching visual navigation technologies with robustness and real-time capabilities has become one of the key technical pathways for advancing the intelligent development of agricultural machinery.

Visual-based crop row centerline detection typically involves two steps: identifying crop rows and estimating crop row centerlines. In the process of identifying crop rows, traditional methods involve segmenting images and backgrounds using color indices and thresholds to extract green vegetation such as weeds and crops from the image, resulting in a binary image. Traditional crop row detection typically relies on color indexes such as the excess green index and threshold segmentation using the Qtsu segmentation algorithm to generate a binary mask for navigation line fitting [3,4,5,6,7,8]. Some studies have incorporated IMU data or have adapted to furrows to improve robustness [9,10]. In summary, traditional image processing methods for crop rows are mostly targeted at the seedling stage of crops. During the early stages of crop growth, weeds that are similar in color to crops compete for growth, limiting the generalization ability of traditional methods based on the Excess Green color index and Otsu’s algorithm. These methods are also easily affected by lighting conditions and weeds in agricultural environments, resulting in poor processing accuracy for situations such as row-to-row occlusion and curved crop rows. making it difficult for them to operate stably and reliably in complex, dynamic field environments over the long term.

In recent years, deep learning technology has been widely applied in various areas of agricultural research, with the identification and detection of crop row features becoming an increasingly significant area of focus. Despite the nascent state of research in this domain, several studies have successfully integrated deep learning methodologies into the realm of crop row detection tasks. The efficacy of these methodologies is evidenced by their capacity for enhanced robustness, generalisation, and dynamic adaptability, as demonstrated by the utilisation of end-to-end learning, automatic feature extraction, and multi-sample training. Consequently, these methods have yielded novel solutions for agricultural visual navigation. Research in this domain is generally categorized into object detection and semantic segmentation. In the realm of object detection, various YOLO-based frameworks have been deployed to localize crop features using bounding boxes. Studies leveraging YOLOv5 [11,12], YOLOv8 [13,14], YOLOX-Tiny [15] and YOLOv10 [16] have achieved high inference speeds, with some systems reaching up to 61.47 FPS or processing times as low as 26 ms [17]. These models have demonstrated precise navigation in specific scenarios like vineyards or cornfields, maintaining heading errors within 2°. Despite their real-time efficiency, YOLO-based models often struggle with the precision required for densely packed rows and fail to provide detailed pixel-level morphological information such as specific crop area and shape data. To address these limitations, semantic segmentation methods have been extensively researched for providing granular, pixel-level classifications. Architectures based on UNet [18,19], ENet [20,21], and InstaCropNet [22] have been improved to handle challenges like undulating terrain, varying illumination, and complex occlusion. For example, studies incorporating instance segmentation, ResNet encoders, or adaptive perspective transformations have achieved high segmentation accuracies exceeding 94%. Specialized models like CDSC-DeepLabV3+ have even reached an mIoU of 94.71% by integrating semantic enhancement modules [23]. Although existing semantic segmentation-based methods offer high precision, their complex network architectures frequently lead to significant computational overhead and slow inference speeds on resource-constrained embedded devices. Furthermore, many current models lack efficient utilization of spatial information and inter-channel communication, limiting their adaptability to diverse agricultural environments. To bridge this gap, this study proposes an improved SAC-DeepLabV3+ method that leverages a lightweight backbone and optimized attention mechanisms to achieve high-precision, real-time crop row recognition.

Current research on agricultural visual navigation is advancing toward greater adaptability to diverse crops and multi-scenario conditions, as well as achieving high precision and lightweight deployment. Although existing methods have demonstrated superior performance under specific conditions, there remains considerable room for improvement in multi-crop integration, complex terrain handling, and navigation behavior decision-making. To address these challenges, we propose a robust and lightweight maize crop row detection method based on an improved DeepLabV3+ architecture (SAC-DeepLabV3+). By integrating specific structural optimizations, this approach extracts crop feature anchor points, clusters them using a DBSCAN-based strategy, and fits navigation lines via a RANSAC-based outlier rejection mechanism to achieve high stability across diverse field layouts. The primary objectives of this study are to: (1) design a lightweight semantic segmentation architecture capable of real-time inference and suitable for edge-device deployment on agricultural machinery; (2) enhance the network’s spatial feature extraction capabilities to precisely focus on crop regions while suppressing visually similar background interference from weeds; and (3) develop a robust geometric post-processing pipeline that can accurately extract navigation lines even under challenging field conditions such as missing seedlings and curved row patterns.

The remainder of this paper is organized as follows: Section 2 details the hybrid dataset construction, the proposed SAC-DeepLabV3+ architecture, and the formalized navigation line extraction methodology. Section 3 presents the experimental results, including ablation studies, comparative analysis with mainstream models, and a quantitative evaluation of navigation accuracy. Section 4 discusses the findings within the context of recent advancements and outlines the future roadmap. Finally, Section 5 draws the conclusions of this study.

## 2. Materials and Methods: From Dataset Construction to the SAC-DeepLabV3+ Recognition Model

**Figure 1:**
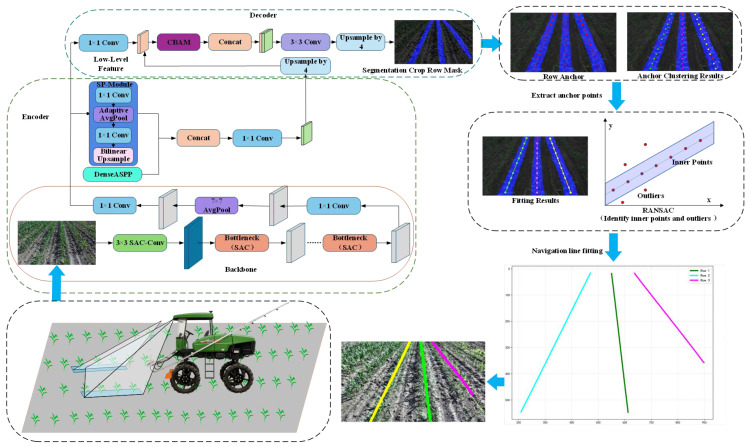
The overall workflow diagram of the proposed SAC-DeepLabV3+ method, illustrating the pipeline from image acquisition and semantic segmentation to anchor point extraction and final navigation line fitting.

This study proposes a method to obtain maize crop row lines through crop row segmentation and navigation line extraction. Crop row detection uses an improved DeeplabV3+ segmentation model to generate a segmentation mask for the crop rows. Then, crop anchor points are extracted from the crop rows, and RANSAC is used to fit the anchor points to obtain accurate crop row lines. The entire workflow is shown in Figure 1.

### 2.1. Dataset Construction and Data Processing

Section 2.1 details the systematic approach to constructing a robust dataset for maize crop row detection. This includes the initial acquisition of raw images from the CRBD benchmark, the strategic application of data augmentation techniques to ensure environmental adaptability, and the rigorous manual annotation process used to establish high-precision semantic labels.

#### 2.1.1. Hybrid Dataset Construction

**Figure 2:**
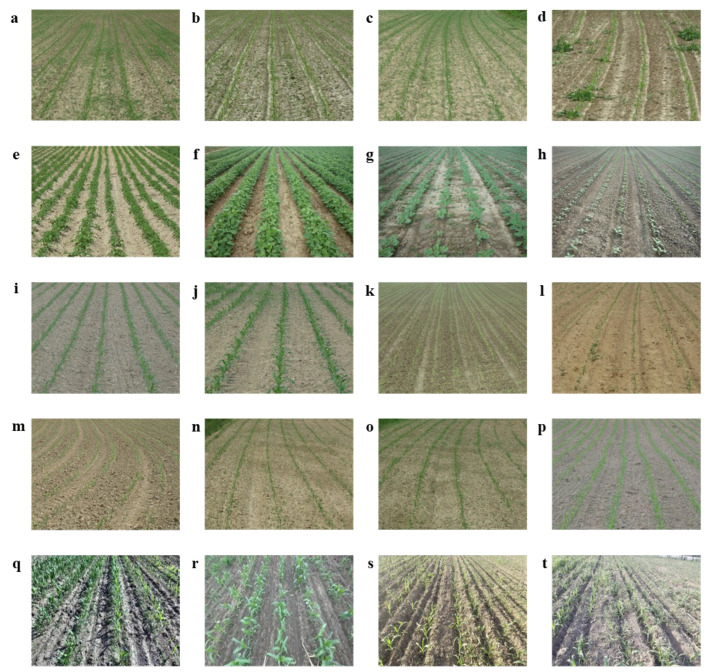
Representative samples from the constructed hybrid dataset used for model training and evaluation. (**a**–**d**) Field scenes severely occluded by weed interference and shadow variations; (**e**–**h**) Diverse crop types showcasing potatoes, soybeans, sunflowers, and onions; (**i**–**l**) Distinct morphological characteristics of crops across different growth stages; (**m**–**p**) Complex unstructured environments featuring curved and irregular crop rows; (**q**–**t**) Target real-field maize seedlings at the 3–5 leaf stage acquired in Gu’an City, demonstrating standard planting patterns.

To comprehensively evaluate the proposed method and ensure its adaptability to complex field environments, a hybrid dataset was constructed. This dataset combines a public benchmark with newly acquired real field data, comprising a total of 834 original images. The first component of the hybrid dataset utilizes the public Crop Row Benchmark Dataset (CRBD) [24], which consists of 281 high-resolution images. These images were acquired in the spring of 2014 in Slavonia, Croatia, using a Panasonic LUMIX DMC-F2 digital camera with moderate variations in yaw, pitch, and roll angles. As illustrated in Figure 2, this subset provides substantial diversity, encompassing complex scenarios such as severe weed interference, distinct crop types (including potatoes, soybeans, sunflowers, and onions), varying morphological characteristics across different growth stages, and curved crop rows. To further validate the algorithm’s robustness under specific local agronomic conditions and expand the dataset volume, a second component of real field data was acquired in Gu’an City, Hebei Province, China. This acquisition contributed an additional 553 images of maize seedlings at the critical 3–5 leaf stage, which is the primary period for inter-row mechanical weeding and spraying. These target maize fields were characterized by a standard row spacing of 60 cm, a plant spacing of 28 cm, sparse weed distribution, and natural lighting variations. To provide a detailed statistical profile of the hybrid dataset, the category distribution across the 834 original images was analyzed. Maize constitutes the absolute majority (553 images, ~66.3%), followed by celery (10.2%), potatoes (8.5%), and onions (6.1%). Other crop types, including sunflowers and soybeans, account for the remaining 8.9% of the total samples. By combining these two distinct data sources, the diversity of feature information is ensured, enabling the model to learn and adapt to various unstructured agricultural scenarios.

Prior to model training, a standardized preprocessing pipeline was uniformly applied to all 834 images in the hybrid dataset to ensure computational efficiency and feature stability. First, the original high-resolution images were resized to 512 × 512 pixels using bilinear interpolation to reduce the memory footprint. Second, pixel intensity values were normalized from the [0, 255] scale to a [0, 1] range to accelerate model convergence. Finally, to mitigate the impact of varying field illumination and sensor noise, the input channels were standardized using the ImageNet dataset’s mean and standard deviation. This standardization strictly aligns the color distribution, enabling the network to extract features robustly across diverse lighting and soil background conditions.

#### 2.1.2. Dataset Partitioning and Data Augmentation

**Figure 3:**
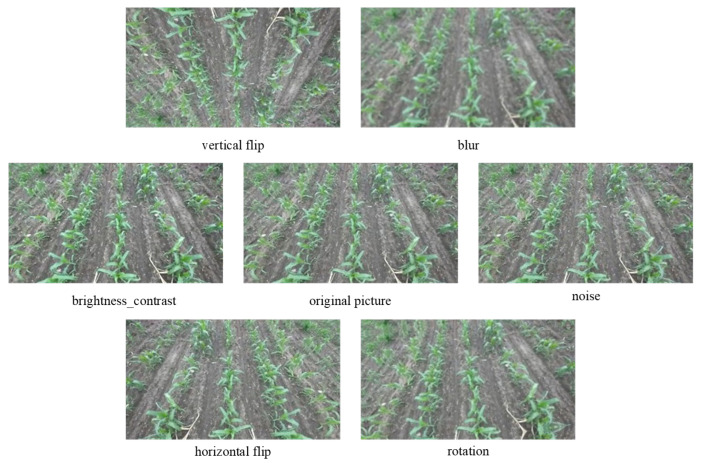
Data enhancement effect diagram.

To ensure the natural expression of image features, further improve the model’s generalization ability and robustness, and reduce overfitting, a systematic data augmentation strategy was implemented on the original dataset, as shown in Figure 3. Image transformation techniques such as brightness and contrast adjustment, horizontal flipping, vertical flipping, blurring, noise addition, and rotation were used. The dataset’s size was expanded to 5000 images, effectively enhancing the model’s ability to cope with complex farmland environments. The original images and enhanced samples are shown in the figure. The enhanced dataset is divided into training, validation, and test sets in an 8:1:1 ratio to ensure the scientific nature of the model’s training and evaluation.

**Figure 4:**
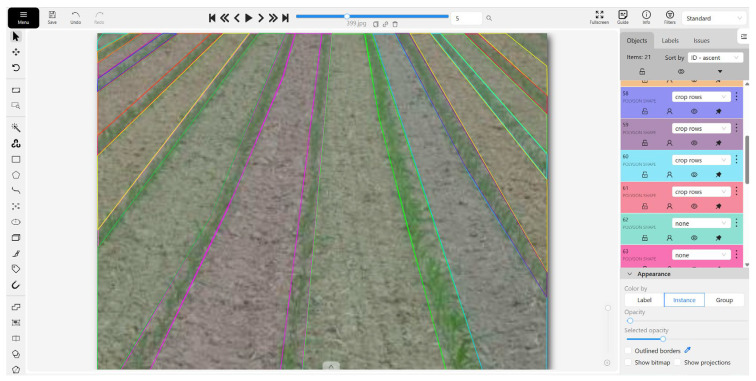
Illustration of the manual pixel-level data annotation process using the CVAT.ai online platform (version 2.12.0). The central workspace displays the polygon-based delineation of crop rows against the soil and weed background. The right-side panel details the annotation configuration tools and object properties, specifically showing the ‘Objects’ list where distinct polygon shapes are assigned semantic labels. Additionally, the panel provides layer visibility toggles, locking mechanisms, and appearance settings, which collaboratively ensure strict quality control and high-precision boundary demarcation for the semantic segmentation model.

As detailed in Algorithm 1, the data augmentation was performed strictly on the training set after the initial 8:1:1 split of the raw dataset. This approach prevents data leakage and ensures that the validation and test sets remain composed of original, unseen images, thereby providing a more objective evaluation of the model’s generalization capabilities in unstructured agricultural environments.
**Algorithm 1** Data Enhancement PipelineInput: Original collected dataset $D_{raw}$; Target total samples N=5000; Split ratios (8:1:1).Output: Augmented training set $D_{train}$, validation set $D_{val}$, and test set $D_{test}$.1. Dataset Splitting:            Split $D_{raw}$ into $D_{train\_raw}$, $D_{val}$, and $D_{test}$ based on the 8:1:1 ratio.            Note: To avoid data leakage, augmentation is only applied to the training set.2. Initialize $D_{train}\leftarrow D_{train\_raw}$
3. while $|D_{train}|<N\times 0.8$ do4.          Randomly select an image-mask pair (I,M) from $D_{train\_raw}$.5.          Stochastic Transformation (Apply one or more operations based on Figure 4):             -Brightness_Contrast: Adjust α∈[0.8,1.2].              -Horizontal/Vertical Flip: 50% probability.             -Rotation: Random angle $\theta \in [-15^{{}^{\circ}},15^{{}^{\circ}}]$.             -Blurring: Apply 3×3 Gaussian kernel.             -Noise Injection: Add Gaussian noise $\sigma^{2}=0.01$.6.          Generate transformed pair $\left(I^{\prime },M^{\prime }\right)$.7.          $D_{train}\leftarrow D_{train}\cup \{(I^{\prime },M^{\prime })\}$.8. end while9. return $D_{train},D_{val},D_{test}$.

#### 2.1.3. Data Labeling and Quality Control

To construct a high-quality semantic segmentation dataset, all images in the CRBD underwent meticulous pixel-level annotation using the CVAT online platform. The detailed workflow and an example of the resulting annotation mask are illustrated in Figure 4. The annotation process established two mutually exclusive semantic categories: “crop rows” (foreground) and “none” (background). Specifically, the “crop rows” category was defined by outlining the complete canopy area of the plants in each row using polygon tools, ensuring that the boundaries strictly adhered to the outermost leaves to provide precise spatial information for the segmentation model. The “none” category included all other image elements, such as soil, stones, and shadows.

To ensure the scientific rigor of the ground truth, three principles were followed during the labeling process: (1) Consistency: The same boundary definition was applied across different growth stages and lighting conditions to prevent the model from learning environmental biases. (2) Detail Retention: Small gaps between plants within the same row were labeled as “Non-Crop Rows” to help the model learn the texture of discontinuous rows. (3) Double Verification: Each labeled image was cross-checked by another researcher to minimize human error and ensure that the annotation quality laid a solid foundation for high-precision navigation line extraction.

### 2.2. Crop Row Recognition Model Based on Semantic Segmentation

#### 2.2.1. Overall Methodology

The proposed methodology follows a hierarchical vision-based pipeline designed for real-time agricultural navigation. It begins with image acquisition and data augmentation to ensure environmental robustness. The core consists of a lightweight SAC-DeepLabV3+ semantic segmentation model for pixel-level crop row identification. Finally, a geometric post-processing stage involving DBSCAN clustering and RANSAC fitting is executed to transform segmentation masks into precise navigation lines.

#### 2.2.2. SAC-DeeplabV3+ Semantic Segmentation Model

DeeplabV3+ is a classic semantic segmentation model that adopts an encoder–decoder structure. This paper improves upon the traditional DeeplabV3+ model. First, a lightweight MobileNetV2 network is used to replace the main trunk extraction network Xception, enhancing the model’s feature extraction capabilities. At the same time, Split-Attention Convolution is used to replace the 3 × 3 Depthwise convolution in the Bottleneck, improving local attention modeling capabilities. Secondly, by modifying ASPP to the DenseASPP + SP module, the model can effectively utilize the global information of the image during multi-scale feature extraction of crop rows, thereby enhancing the model’s ability to identify crops in complex agricultural scenarios. The SP strips achieve a larger and more dense expansion rate through horizontal and vertical strip pooling, capturing long-distance relationships between isolated regions and extracting contextual information. The feature maps obtained by using strip pooling instead of global pooling in the backbone network retain more detailed information. Then, the CBAM attention mechanism is introduced into the decoder to enhance the model’s accurate perception of crop row boundaries, adaptively capture features of different scales, effectively suppress interference such as weeds and missing seedlings, and improve the segmentation accuracy of crop rows.

**Figure 5:**
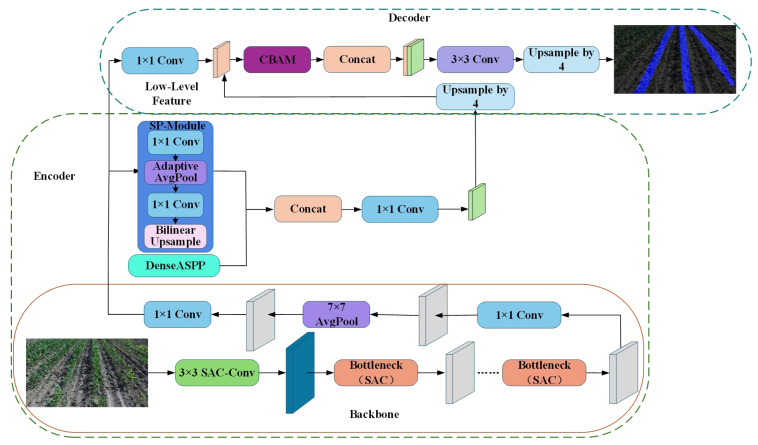
The overall architecture of the proposed SAC-DeepLabV3+ semantic segmentation model. The encoder utilizes a lightweight MobileNetV2 backbone integrated with Split-Attention Convolution (SAC) for robust feature extraction, followed by a DenseASPP + SP module to capture multi-scale contextual information. The decoder employs a Convolutional Block Attention Module (CBAM) to refine spatial and channel features before the final upsampling stage generates the predicted mask.

As shown in Figure 5, the input image in the encoder undergoes feature extraction through a lightweight backbone network, which divides the features into shallow features and deep features. Among these, the shallow features enter the decoder structure 1 × 1 Conv to increase the number of feature channels for the shallow features. Deeper features are obtained at various scales through the DenseASPP + SP module and then fused. Subsequently, a 1 × 1 convolution is applied to reduce the number of feature channels. Meanwhile, deep semantic features are quadruple-upsampled to match the height and width of shallow features, facilitating their integration with the reduced shallow semantic features. Deep features are merged with shallow features. After CBAM-enhanced attention, the merged features undergo 3 × 3 convolution feature compression and quadruple-upsampling to produce a final prediction of the same size as the original image.

#### 2.2.3. Improved MobileNetV2 Backbone Network

The backbone network of the DeeplabV3+ original model is Xception [25], which has high accuracy but a large number of parameters. When deployed on mobile devices, it can easily lead to long inference times, making it impossible to process input image information in real time and affecting the efficiency of the device.

**Figure 6:**
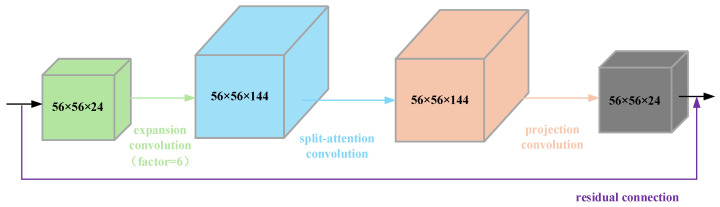
Detailed structure of the SAC-Bottleneck module. By replacing the traditional 3 × 3 depthwise convolution with a Split-Attention Convolution (SAC) mechanism, the network enhances its ability to model local feature dependencies and suppress irrelevant background noise while maintaining a lightweight parameter footprint.

In response to issues such as the high computational cost and susceptibility to overfitting the backbone network Xception, it was replaced with the lightweight network MobileNetV2. This replacement reduces the computational complexity and memory usage of the model while maintaining semantic segmentation accuracy. MobileNetV2 adopts an inverted residual structure, which consists of three core components: First, 1 × 1 convolution is used for channel dimension expansion (expansion layer), followed by efficient feature extraction through 3 × 3 separable convolution (depth convolution layer), and finally dimension reduction (projection layer) through 1 × 1 convolution. The output is then connected to the original input through residual connection, thereby enhancing the model’s nonlinear expression capabilities and avoiding the loss of information. This structure not only significantly reduces the number of parameters and computations, but also occupies less memory and performs faster inference when deployed on mobile devices, while also possessing strong feature extraction capabilities. It effectively improves the running efficiency of the model, alleviates overfitting issues, and can accurately extract the details and structural features of complex crop objects such as corn rows. The spatial channels in MobileNetV2 features have weak interactions. To enhance the model’s ability to perceive textures, boundaries, and other regions in the early stages of feature extraction, Split-Attention Convolution is integrated into MobileNetV2, replacing the 3 × 3 Depthwise convolution in the Bottleneck, as shown in Figure 6. This improvement makes the features of the entire backbone network more discriminative while maintaining the simplicity of the network structure and end-to-end training consistency.

The structural parameters of the improved MobileNetV2 backbone are detailed in Table 1. In this architecture, the Expansion Factor represents the multiplicity of the 1 × 1 convolutional upscaling within the inverted residual structure. The Output Channels indicate the depth of the resulting feature matrix. The Repetition Count denotes the number of times a specific bottleneck layer is repeated, and the Stride defines the step size of the convolution operation.

#### 2.2.4. DenseASPP + SP Module

**Figure 7:**
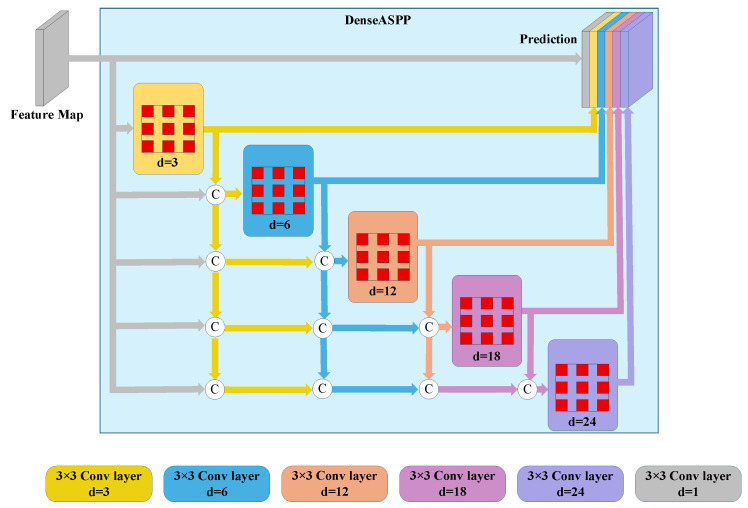
Architecture of the DenseASPP + SP module. This component connects multiple atrous convolutions (with dilation rates of d = 3, 6, 12, 18, 24) in a dense configuration alongside horizontal and vertical strip pooling, effectively extracting dense multi-scale features and preserving long-range contextual dependencies without significant information loss.

Since the Deeplabv3+ network uses ASPP to extract a limited number of pixels at once, ASPP lacks sufficient feature resolution along the scale axis, resulting in significant information loss. To address this issue, this study employs DenseASPP and introduces an SP pooling module to construct the DenseASPP + SP module with a larger receptive field. Both modules adopt a parameter-sharing mechanism, enabling them to use the same parameters. They capture features at various scales by applying zero convolutions across different receptive fields, then fuse these features and pass them to the next layer via dense connections, as is shown in Figure 7, where c denotes the contact connection operation. This network allows six feature layers of different scales to be connected in parallel. First, the input deep features are passed to the output position, followed by successive dilated convolutions with dilation factors of 3, 6, 12, 18, and 24. Each dilated convolution layer takes as input the concatenation of the outputs from all previous convolution layers and the initial input features, thereby generating features of different scales. This enables DenseASPP + SP to effectively utilize features of various scales, more efficiently processing details and contextual information in images. This reduces the need for sample training data, lowers the risk of overfitting, enhances the model’s robustness and stability, and ultimately improves overall segmentation accuracy.

The expression for DenseASPP is

(1) $$y_{l}=H_{k,d_{l}}\left(\left[y_{l-1},y_{l-2},\ldots ,y_{0}\right]\right),$$

where $d_{l}$ is the expansion rate of layer l; $\left[\ldots \right]$ indicates a concatenation operation; and $\left[y_{l-1},y_{l-2},\ldots ,y_{0}\right]$ represents the connection of outputs from all previous layers. Compared to the original ASPP module, DenseASPP stacks all the dilated convolutions and creates dense connections.

#### 2.2.5. CBAM Attention Mechanism

**Figure 8:**
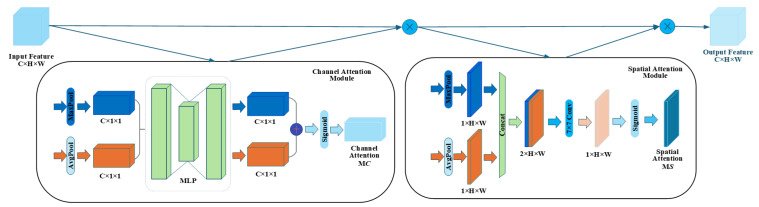
Structure of the Convolutional Block Attention Module (CBAM) integrated into the decoder. It sequentially infers attention maps along the channel and spatial dimensions, enabling the segmentation model to adaptively focus on the fine-grained boundaries of crop rows while effectively suppressing visual interference from weeds.

The fused multi-scale features are then fed into the CBAM attention mechanism module, as shown in Figure 8. CBAM sequentially applies channel attention and spatial attention to the feature maps, performing fine-grained weighting optimization to significantly enhance the network’s ability to focus on critical target regions. Finally, the feature maps optimized by CBAM are sent to the first upsampling module, entering the decoding path to gradually restore spatial resolution and achieve precise image segmentation.

In the channel attention module, the input feature maps are subjected to global average pooling and max pooling operations, respectively, to generate two channel description vectors, which are then input into a shared multi-layer perceptron (MLP) to calculate the attention weights for the channel dimension. This weight vector is multiplied channel-by-channel with the original feature map to obtain the channel-weighted feature map, effectively emphasizing channel information that is more sensitive to the task. Subsequently, the channel-weighted features are input into the spatial attention module. This module first performs max pooling and average pooling operations on feature maps in the channel dimension to generate two spatial description maps. These two maps are then concatenated and fused through a standard convolution layer to extract a spatial attention map. Finally, this spatial weight map is multiplied element-wise with the input feature map to generate a spatially optimized feature map, thereby precisely guiding the model to focus on key spatial locations in the target area.

The CBAM can adaptively adjust the representation of feature maps without significantly increasing computational overhead, enhancing the model’s ability to perceive local details such as crop row boundaries and textures. By introducing an attention mechanism, the model can more effectively distinguish between targets and backgrounds when faced with complex field environments, such as changes in lighting, background interference, and intermingled crops and weeds, significantly improving overall segmentation accuracy and environmental adaptability. In addition, the attention mechanism helps the model selectively ignore irrelevant information when faced with agricultural images containing unlabeled objects or background interference areas, further improving the accuracy and stability of crop row recognition. The channel attention mechanism is shown in Equation (2), and the spatial attention mechanism is expressed in Equation (3).

(2) $$\begin{array}{l} M_{c}=Sigmoid\left(MLP\left(AvgPool\left(F\right)\right)+MLP\left(MaxPool\left(F\right)\right)\right)\\ \;\;=Sigmoid\left(W_{1}\left(W_{0}\left(F_{avg}^{c}\right)\right)+W_{1}(W_{0}(F_{\max}^{c}))\right) \end{array},$$

(3) $$\begin{array}{l} M_{s}=Sigmoid(conv(\left[AvgPool(F);MaxPool(F)\right]))\\ \;\;=Sigmoid(conv()\left[F_{avg}^{c},F_{\max}^{c}\right]) \end{array}.$$

In the formula, F—Input feature map; $AvgPool\left(F\right)$—Global average pooling; $MaxPool\left(F\right)$—Global max pooling; Sigmoid(•)—Element-wise sigmoid activation, compressing values to the (0, 1) interval; and $F_{avg}^{c},F_{\max}^{c}$—Perform average/maximum pooling on channel dimensions.

### 2.3. Model Evaluation Indicators

To comprehensively evaluate the performance of semantic segmentation models in agricultural machinery visual navigation tasks, this study employed multiple evaluation metrics, including Mean Pixel Accuracy (mPA), Mean Intersection over Union (mIoU), Precision, Recall, *f*1-score, Dice Coefficient, and Overall Accuracy. All these metrics are based on the confusion matrix.

**Figure 9:**
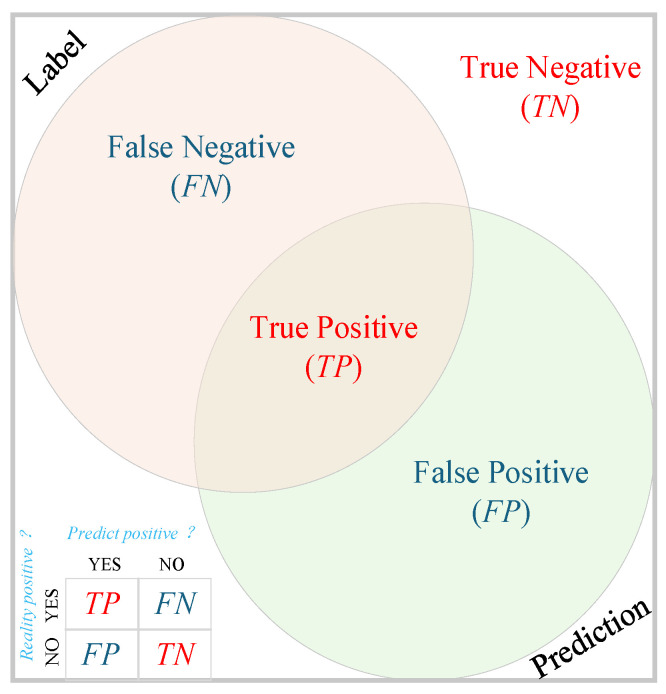
Accuracy calculation indicator relationship diagram.

**Figure 10:**
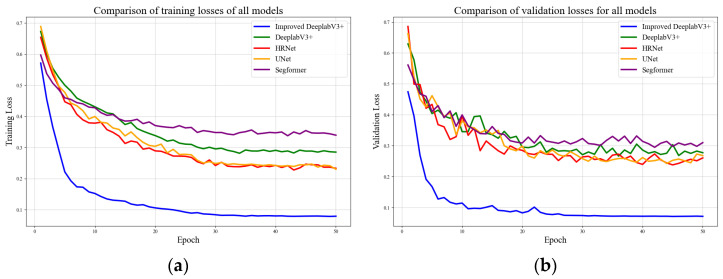
Loss function. (**a**) Training loss function; (**b**) Validation loss function.

This study focuses on binary classification problems and uses a binary confusion matrix to evaluate model performance. As shown in Figure 9, the confusion matrix includes four key metrics: ① True Positive (TP): The model correctly identifies crop row areas as crop rows. ② False Positive (FP): The model incorrectly identifies non-crop row areas as crop rows. ③ False Negative (FN): The model incorrectly identifies non-crop row areas as crop rows. ④ True Negative (TN): The model correctly identifies non-crop row areas as non-crop rows. As shown in Figure 10, the circle on the left represents the set of samples with true labels as crop rows, and the circle on the right represents the set of samples predicted by the model as crop rows. The intersection of the two sets is the true positive (TP), while the other areas represent false negatives (FN), false positives (FP), and true negatives (TN), respectively. This visualization method intuitively reveals the relationship between the model’s predictions and the true labels, aiding in a deeper understanding of the practical significance of each evaluation metric.

Measures the ratio of the intersection and union of the model prediction area and the actual annotation area. It is defined as

(4) $$IoU=\frac{TP}{TP+FP+FN},\;mIoU=\frac{1}{N}\sum_{i=1}^{N}IoU_{i}.$$

Accuracy refers to the proportion of true positive samples in the results, reflecting the accuracy of the classification results:

(5) $$Precision=\frac{TP}{TP+FP}\times 100\%.$$

The recall rate (R) quantifies the proportion of positive samples correctly retrieved out of all positive samples, reflecting the completeness of the classification results:

(6) $$Recall=\frac{TP}{TP+FN}\times 100\%.$$

Precision measures how many of the predicted positive samples are actually true positives, while Recall reflects the proportion of all positive samples that are correctly predicted.

Mean Pixel Accuracy (mPA) represents the average pixel classification accuracy for each category:

(7) $$mPA=\frac{1}{N}\sum_{i=1}^{N}\frac{TP_{i}}{TP_{i}+FN_{i}}.$$

The *f*1-score is the harmonic mean of accuracy and recall, which balances the evaluation of accuracy and completeness:

(8) $$f1-score=\frac{2TP}{2TP+FP+FN}.$$

Since precision (P) and recall (R) are complementary, this paper uses the harmonic mean *f*1 value of the two to evaluate the experimental results.

## 3. The Mathematical Analysis of the Results

### 3.1. Model Training

#### 3.1.1. Experimental Platform and Parameter Settings

During model training, this study used the Adam optimizer to minimize the loss function, setting the momentum factor to 0.9 and the initial learning rate to 1 × 10^−3^. The learning rate was then adjusted adaptively based on batch size using a cosine annealing strategy to achieve dynamic learning rate adjustment. To suppress model over-fitting, a weight decay mechanism was introduced, with the weight decay coefficient set to 5 × 10^−4^. The model training was conducted for a total of 50 epochs of end-to-end training, with a training batch size of 8. To improve the model’s learning ability under small sample conditions, a transfer learning strategy was used to initialize the network weights, accelerating model convergence and enhancing segmentation performance. All training and validation in this experiment were completed on the experimental platform listed in Table 2.

#### 3.1.2. Loss Function

To address the extreme foreground-background class imbalance and refine the segmentation boundaries of crop rows, a combined loss function integrating Focal Loss and Dice Loss was employed. The total loss $L_{total}$ is defined as the weighted sum of both components:

(9) $$L_{total}=L_{focal}+L_{dice},$$

where $L_{focal}$ focuses on hard-to-classify examples and $L_{dice}$ optimizes the global pixel-level intersection over union. The individual formulas are expressed as follows:

(10) $$L_{focal}=-\alpha_{t}{\left(1-p_{t}\right)}^{\gamma }\log(p_{t}).$$

In the formula, γ—Adjusting the focus factor for difficult samples; α—Category weighting coefficients when categories are unbalanced.

(11) $$L_{dice}=1-\frac{2\left|A\cap B\right|}{\left|A\right|+\left|B\right|}.$$

In the formula, *A*—Prediction area; *B*—Real area; and |*A*∩*B*|—Number of pixels in crop rows.

A comparison of the loss convergence between the proposed model and the baseline networks is presented in Figure 10. As shown in Figure 10a, the training loss of the improved DeepLabV3+ exhibits a rapid decrease within the first 10 epochs and subsequently stabilizes at approximately 0.08 after 50 epochs, which is significantly lower than that of the other models. As shown in Figure 10b, the validation loss curve follows a similar downward trend and ultimately stabilizes at approximately 0.09. This overall smoothness in both curves reflects a highly stable gradient update process and indicates that the improved network structure can efficiently learn key features in the early stages. Most notably, the difference between the training loss and validation loss of the proposed model ultimately has a generalization gap of less than 0.01. In contrast, the baseline models typically exhibit a much wider gap ranging from 0.03 to 0.06. This minimal generalization gap confirms that the model successfully avoids overfitting and performs highly reliably on unseen samples. In summary, the proposed method outperforms the baselines in terms of convergence speed, minimum loss level, stability, and generalization capability, fully demonstrating the effectiveness of the optimized architecture.

#### 3.1.3. Ablation Test

This study conducted systematic ablation experiments, as shown in Table 3. Using DeepLabV3+ and Xception as the base model, we evaluated the independent and synergistic contributions of each improvement to the overall performance. Let A denote replacing Xception with MobileNetV2 in the backbone; B denotes replacing the 3 × 3 DW Conv with SAC, C denotes replacing the original ASPP with DenseASPP + SP, and D denotes introducing the CBAM attention mechanism.

**Figure 11:**
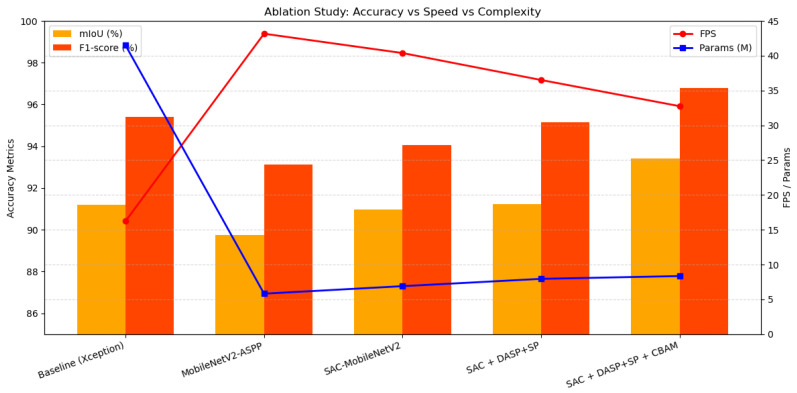
Visual representation of the ablation experiment results, demonstrating the trade-offs between segmentation accuracy, inference speed, and model complexity. The bar chart compares the mIoU and *f*1-score across different structural iterations, while the line graphs track the corresponding changes in frames per second (FPS) and the total parameter count (M).

As shown in Table 3, the improved DeepLabV3+ model in this paper achieves an mIoU of 93.42% and an *f*1-score of 96.8%, both of which are higher than the values of the previously improved models, demonstrating the outstanding performance of the improved model in terms of object detection accuracy and reliability. In terms of model parameter count and computational requirements, the improved full DeepLabV3+ model has 8.35 million parameters, significantly lower than the original base model’s 41.5 million. The significant reduction in model parameters provides a clear advantage in terms of model lightweighting, with the FPS maintained at 32 frames per second, meeting the real-time navigation requirements for high-clearance agricultural sprayers in corn rows. As shown in Figure 11, in the DeepLabV3+ framework, using MobileNetV2 as the backbone network and introducing modules such as DenseASPP + SP and CBAM can significantly enhance the performance metrics of the object detection model, such as mIoU and *f*1-score, while effectively reducing the number of model parameters. The results show that the improvements made demonstrate high efficiency and performance, making them suitable for deployment on resource-constrained embedded platforms or agricultural machinery edge devices.

### 3.2. Analysis of Experimental Results

**Figure 12:**
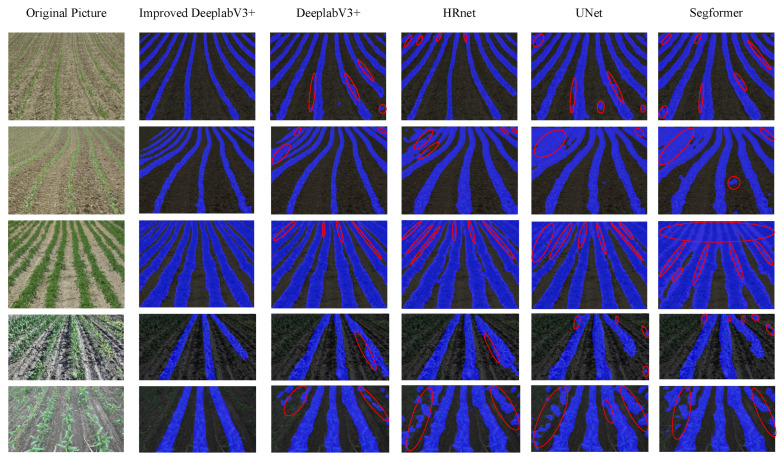
Qualitative comparison of crop row segmentation results across different state-of-the-art models (Improved DeepLabV3+, original DeepLabV3+, HRNet, UNet, and SegFormer) in varying complex field scenarios. The proposed model demonstrates superior robustness in maintaining continuous crop row boundaries under severe weed interference, shadows, and curved planting patterns.

The qualitative segmentation results of the improved DeepLabV3+ model are compared with those of the baseline DeepLabV3+, HRNet, UNet, and SegFormer models, as is visually presented in Figure 12. The quantitative metrics, including Precision, mIoU, mPA, Recall, and Average Inference Time (AIT) for the different models, are summarized in Table 4. To accurately reflect the real-time processing capabilities of the models under normal deployment conditions, a rigorous measurement protocol was implemented for the AIT. Due to operations such as model initialization and GPU memory allocation, the initial inference cycles experience significant latency overhead. To eliminate this bias, a 10-frame warm-up phase was executed prior to measurement. Subsequently, the AIT was calculated by averaging the processing time of 100 consecutive frames, utilizing torch.cuda.synchronize() to ensure precise GPU timing. It is important to note that the AIT reported in Table 4 strictly measures the neural network’s forward pass and explicitly excludes the post-processing navigation line extraction time.

To comprehensively evaluate the segmentation performance of the proposed model, it was compared with current mainstream semantic segmentation networks (including DeepLabV3+, HRNet, UNet, and SegFormer) under identical experimental conditions. As shown in the comparison results, the proposed model achieves a highly competitive balance across key evaluation metrics, with Precision, mIoU, mPA, and Recall reaching 99.5%, 87.6%, 96.5%, and 96.7%, respectively. Compared to high-precision baseline models such as the original DeepLabV3+ and HRNet, the proposed method not only yields higher accuracy but also substantially reduces the average inference time (AIT to 19.3 ms), demonstrating strong feasibility for real-time applications. Conversely, when compared to lightweight networks such as UNet and SegFormer, the proposed method achieves noticeably higher segmentation robustness while maintaining a comparable inference speed. These findings indicate that the proposed model provides a strong comprehensive advantage in crop row recognition tasks, effectively balancing the dual requirements of high accuracy and real-time inference for automated agricultural machinery.

A qualitative comparison of the segmentation results is presented in Figure 12, which visually demonstrates the superior robustness of the improved SAC-DeepLabV3+ model across various challenging field scenarios. As highlighted by the red circles in the figure, baseline models such as the original DeepLabV3+, HRNet, UNet, and SegFormer frequently exhibit misclassifications. For instance, in the second and third rows characterized by severe weed interference and curved planting patterns, the baseline models suffer from noticeable false positives, incorrectly segmenting inter-row weeds as crop rows. Additionally, in scenarios with shadows and discontinuous seedlings (rows 4 and 5), the standard UNet and SegFormer produce fragmented and discontinuous masks. In contrast, the proposed method successfully suppresses background noise and accurately delineates clear, continuous crop row boundaries. This visual evidence confirms that the integration of Split-Attention Convolutions and the CBAM effectively enhances the model’s ability to distinguish target crops from visually similar weed backgrounds, ensuring high-quality masks for subsequent navigation line extraction.

### 3.3. Navigation Line Extraction and Evaluation

#### 3.3.1. Navigation Line Extraction

**Figure 13:**
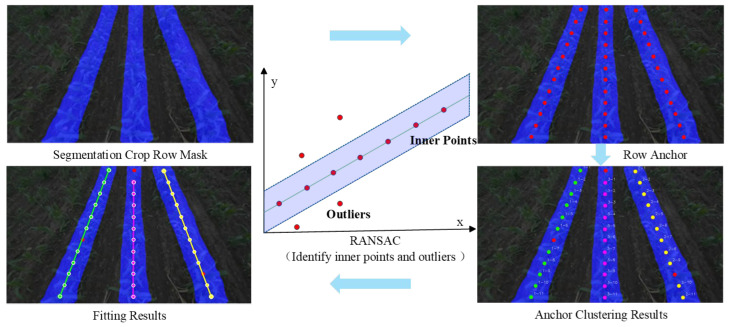
The geometric post-processing workflow for navigation line extraction. The pipeline transforms the continuous semantic segmentation mask into discrete row anchors, separates distinct rows using DBSCAN clustering, and successfully rejects outlier noise points via the RANSAC algorithm to fit highly precise linear trajectories.

When the intelligent sprayer equipped with a forward-facing camera moves through farmland, considering that the agricultural machinery moves at a relatively slow speed, the system should focus more on crop information in the near-field area in front of the machinery. The core area of agricultural machinery movement is set as the forward guidance zone, thereby limiting the maximum number of crop rows, typically 2~4 rows of crops in the center of the camera’s field of view [16]. This study selected the middle three crop rows for analysis. To convert the crop row instances segmented by the improved Deeplabv3+ model into effective information for guiding agricultural machinery navigation, it is necessary to precisely extract the navigation lines from the obtained crop row segmentation masks. The process of extracting navigation lines is shown in Figure 13.

Different crop rows are identified using a horizontal segmentation algorithm. Connected region analysis is performed on each crop mask row. Based on the density distribution of the x-coordinate, the DBSCAN clustering algorithm is used to assign a unique instance identifier. Specifically, the DBSCAN algorithm was empirically configured with a neighborhood radius (*eps*) of 30 pixels and a minimum number of samples (*min_samples*) of 5 to optimally group pixels belonging to the same crop row. Subsequently, morphological closing operations are applied to each instance in the vertical direction to repair broken areas and obtain a coherent and complete row mask. The Canny algorithm is applied to perform edge detection on crop row masks. Through vertical equidistant sampling of anchor points, fragment information of crops in certain crop rows is extracted. Instead of utilizing off-the-shelf object detectors, a mathematical centroid calculation method based on mask moments is then used to obtain the center-of-mass points of the crops. By combining the vertical coordinates of the row anchor points, different coordinate point information for segmenting crop rows is formed, which serves as the feature points of the crops. To address issues such as missing plants or broken rows in crop rows, where pixels are missing at a certain height within a crop row, an up–down search strategy is employed to replace missing pixels with the nearest valid center, thereby enhancing the completeness of the anchor point set. This ensures that each row has at least a sufficient number of anchor points to guarantee the reliability of subsequent fitting.

For all detected anchor points, the Random Sample Consensus (RANSAC) algorithm is used to check the number of interior points in the crop row mask where the distance to the line is less than the threshold, and the solution with the most interior points is selected as the current crop row. Then, a spatial consistency check is performed on the interior points along the line to remove noise points, and the resulting discrete anchor point set is fitted to obtain the precise navigation reference line. Compared with the Hough transform and direct least squares method, the core advantage of the RANSAC algorithm lies in its ability to exclude the interference of abnormal anchor points, enabling accurate fitting of navigation lines even in the presence of weeds, uneven soil, and missing crops. The RANSAC algorithm possesses strong noise resistance and robustness, making it highly suitable for complex agricultural scenarios characterized by high noise levels and numerous outliers. The pseudocode for the RANSAC algorithm is as follows:

The parameters for Algorithm 2 were empirically set as K=100 and T=5 pixels to ensure a balance between computational efficiency and fitting robustness.
**Algorithm 2** RANSAC-based Navigation Line FittingInput: Anchor set $P=\left\{p_{1},p_{2},\ldots ,p_{n}\right\}$; maximum iterations K=100; inlier distance threshold T=5 pixels; minimum inliers required $M_{in}=10$.Output: Optimal navigation line model parameters $\theta_{best}=(a,b,c)$ where ax+by+c=0.1. Initialize: *best_model* ← null, *max_inliers* ← 0, $S_{best}\leftarrow Ø$.2. for *k* = 1 to K do3.     $P_{sample}\leftarrow \mathrm{Select}\;\mathrm{two}\;\mathrm{points}\;\mathrm{randomly}\;\mathrm{from}\;P$.4.     $\theta_{curr}\leftarrow \mathrm{FitLineModel}(P_{sample})$: Solve for (a,b,c) passing through $P_{sample}$.5.     $S_{curr}\leftarrow Ø$
6.     for each $p_{i}(x_{i},y_{i})\in P$ do7.     $d_{i}\leftarrow \frac{\left|ax_{i}+by_{i}+c\right|}{\sqrt{a^{2}+b^{2}}}$: Compute perpendicular distance from $p_{i}$ to $\theta_{curr}$.8.          if $d_{i}<T$ then9.             $S_{curr}\leftarrow S_{curr}\cup \{p_{i}\}$.10.        end if11.     end for12.     if $|S_{curr}|>max\_inliers$ and $|S_{curr}|\geq M_{in}$ then13.         $max\_inliers\leftarrow |S_{curr}|,S_{best}\leftarrow S_{curr},best\_model\leftarrow \theta_{curr}$.14.     end if15. end for16. if best_model≠null then17.     *best_model* ← Least Squares Fit($S_{best}$):Re-estimate model using all inliers in $S_{best}$ via the least squares method to minimize $\sum d_{i}^{2}$.18. end if19. return *best_model.*

#### 3.3.2. Evaluation Results of Navigation Line Extraction

**Figure 14:**
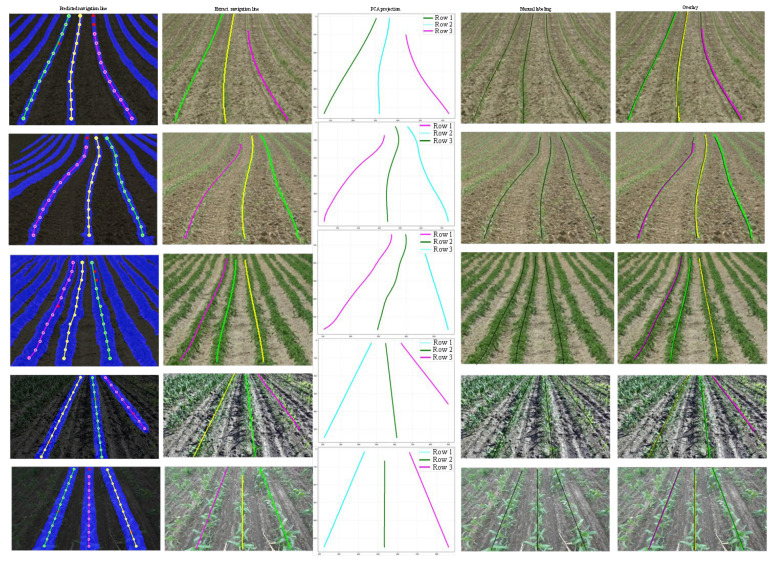
Step-by-step visualization of the navigation line extraction process and qualitative comparison against manual ground-truth labels. In the PCA projection and Overlay subplots, the different assigned colors (e.g., cyan, green, and magenta) do not denote biological crop types; rather, they serve exclusively to visually differentiate the distinct crop row instances as separated by the DBSCAN clustering algorithm.

To intuitively evaluate the navigation line extraction algorithm, Figure 14 visualizes the step-by-step post-processing results. Because each crop row is assigned a unique instance label during the clustering phase, anchor points from different rows are strictly distinguished, preventing cross-interference. The visual results demonstrate the exceptional geometric robustness of the DBSCAN and RANSAC pipeline. In the first three rows featuring complex curved trajectories, the algorithm successfully fits smooth navigation paths that dynamically adhere to the actual curvature of the crop rows. Furthermore, in areas where crop canopy discontinuities or missing seedlings occur, the RANSAC algorithm effectively rejects outlier noise and maintains the correct trajectory trend. It is worth noting that this robustness is particularly pronounced in straight crop rows; even if several row anchors fail to yield predicted points, the trajectory determination remains unaffected, as a straight line can be accurately established with only two valid points within the same instance. Consequently, as observed in Figure 14, the extraction accuracy for straight rows is inherently higher than for curved ones. Nevertheless, the “Overlay” column demonstrates a near-perfect alignment between the algorithmically generated lines (colored) and the manual annotations (black) across all scenarios. This high degree of visual overlap validates that the proposed extraction strategy can provide reliable and highly precise directional guidance for intelligent agricultural machinery, regardless of straight or non-linear field layouts.

To comprehensively evaluate the statistical reliability of the proposed pipeline, the extraction errors at different crop row locations were quantitatively analyzed. As shown in Table 5, the mean and standard deviation of the angular deviation for the middle crop row are 0.94° and 0.20°, respectively. Both metrics are significantly smaller than those of the left crop row (1.41° ± 0.43°) and the right crop row (1.32° ± 0.35°). This is primarily because the camera is typically mounted at the central axis of the agricultural machinery, resulting in the middle crop row being the least affected by lens distortion, parallax, and occlusion. Similarly, the average and standard deviation of the lateral distance deviation for the middle row are 2.1 ± 1.29 pixels, which are vastly superior to the left (4.26 ± 3.52 pixels) and right (3.68 ± 2.35 pixels) rows. The anchor point fitting accuracy for the middle crop row reaches 96.67%, approximately 5 and 7 percentage points higher than the left and right rows, respectively. The consistently low variance observed in the middle row demonstrates the high stability of the RANSAC geometric fitting process. Consequently, the middle crop row is the most suitable and reliable visual navigation reference for high-clearance intelligent sprayers in practical applications.

Furthermore, the average error of the navigation lines obtained in this study was compared with state-of-the-art results reported in the recent literature. For instance, in scenarios involving missing corn seedlings and sparse weeds, Reference [15] reported an average angular error of approximately 2.95°, while Reference [22] reported an average fitting error of 1.71° under varying growth stages and uneven lighting. Compared to these baseline values, the average angular deviation of the middle navigation line in this study is merely 0.94°. This significant reduction in error metrics definitively demonstrates that the proposed SAC-DeepLabV3+ pipeline, coupled with the RANSAC post-processing strategy, achieves superior accuracy, better adapts to complex uncalibrated farmland environments, and fully meets the rigorous precision requirements for autonomous agricultural navigation.

## 4. Discussion

Vision-based navigation line extraction technologies for intelligent agricultural machinery and agricultural robots have attracted significant attention due to their advantages of low cost and system stability. However, most existing computer vision-based navigation line extraction algorithms suffer from large model sizes and poor deployability on hardware devices. In this study, we proposed a crop row detection and navigation line extraction pipeline based on an improved DeepLabV3+ network. Experimental results demonstrate that the proposed approach effectively addresses the accuracy degradation of GNSS-based autonomous navigation under multipath conditions. It provides a reliable, real-time, and precise path navigation solution for intelligent agricultural machinery operating in early-growth-stage fields, ultimately promoting higher levels of agricultural mechanization.

Contextualization and Recent Advancements:

To situate this work within the broader context of vision-based agricultural navigation and address the gaps left by previous studies, the recent literature has increasingly focused on integrating advanced attention mechanisms and lightweight backbones to balance accuracy and edge-device deployability [26,27]. For instance, comparative studies utilizing lightweight architectures like MobileNetV2 combined with semantic segmentation models have shown great promise in accurately distinguishing complex weed backgrounds [28]. Similarly, optimized clustering and geometric fitting algorithms, such as Threshold-DBSCAN coupled with least squares methods, have been recently highlighted for their robustness in multi-crop centerline extraction [29].

Furthermore, state-of-the-art detection architectures have rapidly evolved. Recent studies have demonstrated exceptional performance leveraging Transformer-based architectures like RF-DETR and the latest CNN-based variants like YOLOv12 and YOLO26 [30,31]. Models such as RF-DETR utilize deformable attention for superior global context modeling in complex orchards, while YOLO26 introduces NMS-free inference and advanced label assignment for extreme real-time efficiency on edge devices. While these highly optimized bounding-box-based detection models excel in single/multi-class localization and speed, our semantic segmentation approach (coupled with DBSCAN and RANSAC) offers a distinct advantage: superior pixel-level morphological details. This precise pixel-level contouring is absolutely critical for extracting exact crop canopy centroids and navigating highly curved rows, successfully addressing the morphological and geometrical limitations inherent in purely bounding-box-based detection methods.

Limitations of the Current Study:

Despite the promising results achieved, this study acknowledges certain limitations. First, while our hybrid dataset successfully expanded the training volume to address data scarcity, the data primarily covers the early-to-mid growth stages (3–5 leaf stage) of maize. The model’s performance in highly mature crop fields with severe canopy overlap, which completely obscures inter-row spacing, remains to be further validated. Second, extreme environmental conditions, such as severe motion blur caused by highly uneven terrain, may degrade the quality of the segmentation masks. Finally, the current pipeline relies on sequential steps (segmentation followed by post-processing) and requires extensive pixel-level image annotations, which limits the pursuit of a truly end-to-end extraction process.

Future Roadmap:

Drawing inspiration from the aforementioned architectural enhancements and addressing current limitations, our future research will focus on the following key directions:

(1) Architectural Evolution: We will explore integrating lightweight Transformer-based encoders (similar to the self-attention mechanisms in RF-DETR) into our current lightweight decoder. This will further enhance global context understanding (e.g., mitigating single-frame degradation caused by temporary occlusions) without sacrificing the real-time inference speed demonstrated by our current pipeline.
(2) End-to-End and Universal Applicability: To extend this method to multi-crop and multi-season scenarios, domain adaptation and few-shot learning techniques will be prioritized. Furthermore, taking cues from the end-to-end paradigms of newer YOLO iterations, we will investigate truly end-to-end navigation line extraction models to eliminate cumbersome post-processing steps.
(3) Multi-Sensor Fusion: By integrating vision-based navigation with GNSS, inertial navigation systems (INS), and LiDAR, we aim to overcome the limitations of single-mode navigation under extreme lighting or severe occlusions, providing a highly resilient solution for agricultural robots operating in unstructured field conditions.

## 5. Conclusions

This research developed an intelligent and lightweight crop row detection method to support the autonomous navigation of agricultural machinery in complex field environments. In summary, the primary contributions of this study encompass the design of the highly efficient SAC-DeepLabV3+ architecture and the development of a robust navigation line extraction pipeline. Specifically, a lightweight DeepLabV3+ segmentation model was proposed, integrating a MobileNetV2 backbone where traditional 3 × 3 depthwise convolutions were replaced with Split-Attention Convolutions (SAC) to enhance local feature modeling. By incorporating a DenseASPP + SP module for multi-scale information fusion and a Convolutional Block Attention Mechanism (CBAM) for fine-grained feature weighting, the network significantly improved its focus on key crop row regions while suppressing background interference. Furthermore, a robust post-processing pipeline utilizing DBSCAN clustering and RANSAC geometric fitting was established to ensure reliable directional guidance.

The experimental results demonstrated that this architectural combination achieved a mean Intersection over Union (mIoU) of 93.42% and an *f*1-score of 96.80%, significantly outperforming the baseline DeepLabV3+ and other mainstream models. The optimization strategies effectively reduced the model parameters to 8.35 M—an approximately 80% decrease compared to the 41.5 M of the original Xception-based model. Crucially, the network achieved a pure Average Inference Time (AIT) of 19.26 ms, allowing the entire visual perception pipeline to maintain a real-time processing speed of over 32 FPS. Furthermore, the navigation line extraction pipeline, based on DBSCAN clustering and RANSAC fitting, proved highly robust against noise from weeds and crop gaps, with the middle crop row yielding the highest precision. In summary, this study provides a high-performance and easily deployable visual perception solution that effectively balances segmentation accuracy and computational efficiency, offering strong technical support for automated navigation in precision agriculture.

## Figures and Tables

**Table 1:** Network structure parameters table.

Operator	Expansion Factor	Output Channels	Repetition Count	Stride
SAC-Conv	–	32	1	2
Bottleneck (SAC)	1	16	1	1
Bottleneck (SAC)	6	24	2	2
Bottleneck (SAC)	6	32	3	2
Bottleneck (SAC)	6	64	4	2
Bottleneck (SAC)	6	96	3	1
Bottleneck (SAC)	6	6	160	3
Bottleneck (SAC)	6	320	1	1
Conv2d 1 × 1	–	1280	–	1
Avgpool 7 × 7	–	–	1	–
Conv2d 1 × 1	–	k	–	–

**Table 2:** Experimental platform.

Name	Device Related Configuration
CPU	Intel(R) Core(TM) i7-12800HX@2.50 GHz
GPU	NVIDIA GeForce RTX 4070 Laptop
Main Memory	32 GB
GPU Acceleration Library	CUDA 11.3, CUDNN 8.2.1
Operating System	Windows 11 (64 bit)
Software Environment	Python 3.9, Pytorch 1.7.0

**Table 3:** Ablation experiments.

Method	mIoU (%)	*f*1-Score (%)	Params (M)	FPS/(Frames·s^−1^)
Base	91.18	95.42	41.5	16.23
A	89.75	93.13	5.82	45.16
A + B	90.97	94.06	6.90	40.38
A + B + C	91.23	95.16	7.95	36.51
A + B + C + D	93.42	96.80	8.35	32.75

**Table 4:** Comparison of different semantic segmentation models.

Model	Precision (%)	mIoU (%)	mPA (%)	Recall (%)	AIT (ms)
DeepLabV3+	98.7	83.9	94.5	93.8	71.6
HRNet	98.6	84.7	95.2	92.7	33.3
UNet	96.3	77.6	92.4	91.2	12.4
Segformer	96.2	77.1	91.7	90.2	20.9
Ours	99.5	87.6	96.5	96.7	19.3

**Table 5:** Navigation line errors at different locations.

Crop Row Location	Angle Deviation (°)		Horizontal Distance Deviation (Pixels)		Anchor Point Fitting Accuracy
	Average	Standard Deviation	Average	Standard Deviation	
Left	1.41	0.43	4.26	3.52	91.67%
Middle	0.94	0.20	2.10	1.29	96.67%
Right	1.32	0.35	3.68	2.35	89.29%

## Data Availability

The minimal dataset (hybrid dataset) is fully open access and permanently hosted on Zenodo at: https://doi.org/10.5281/zenodo.18700580 (accessed on 11 May 2026). All author-generated code underpinning the findings in this manuscript is openly available without restrictions on GitHub at: https://github.com/YongzhiCui/CropRow-Navigation (accessed on 11 May 2026).
